# Lipid phosphate phosphatase-3 regulates tumor growth *via *β-catenin and Cyclin-D1 signaling

**DOI:** 10.1186/1476-4598-10-51

**Published:** 2011-05-11

**Authors:** Ishita Chatterjee, Joseph O Humtsoe, Erin E Kohler, Claudio Sorio, Kishore K Wary

**Affiliations:** 1 Dept of Pharmacology, University of Illinois, 835 S Wolcott, Room E403, Chicago, IL 60612, USA; 2 Department of Cell and Tissue Biology, University of California San Francisco, 521 Parnassus 18 Ave., CA 94143, USA; 3 Department of Pathology and Diagnostics, University of Verona, Strada Le Grazie 8, 37134 Verona, Italy

## Abstract

**Background:**

The acquisition of proliferative and invasive phenotypes is considered a hallmark of neoplastic transformation; however, the underlying mechanisms are less well known. Lipid phosphate phosphatase-3 (LPP3) not only catalyzes the dephosphorylation of the bioactive lipid sphingosine-1-phosphate (S1P) to generate sphingosine but also may regulate embryonic development and angiogenesis *via *the Wnt pathway. The goal of this study was to determine the role of LPP3 in tumor cells.

**Results:**

We observed increased expression of LPP3 in glioblastoma primary tumors and in U87 and U118 glioblastoma cell lines. We demonstrate that *LPP3*-knockdown inhibited both U87 and U118 glioblastoma cell proliferation in culture and tumor growth in xenograft assays. Biochemical experiments provided evidence that *LPP3*-knockdown reduced β-catenin, CYCLIN-D1, and CD133 expression, with a concomitant increase in phosphorylated β-catenin. In a converse experiment, the forced expression of LPP3 in human colon tumor (SW480) cells potentiated tumor growth *via *increased β-catenin stability and CYCLIN-D1 synthesis. In contrast, elevated expression of LPP3 had no tumorigenic effects on primary cells.

**Conclusions:**

These results demonstrate for the first time an unexpected role of LPP3 in regulating glioblastoma progression by amplifying β-catenin and CYCLIN-D1 activities.

## Background

The lipid phosphate phosphatases (LPPs) have been shown to dephosphorylate sphingosine 1-phosphate (S1P) and its structural homologs to produce metabolic products such as sphingosine, ceramide, and lysophosphatidic acid (LPA) [1-3]. Three major isoforms, LPP1, LPP2, and LPP3 have been studied in various tissues and cell lines [3,4], however, the role of LPPs in tumor progression is not well understood [1-4]. Although LPPs are localized to the endoplasmic reticulum (ER), cytoplasm and plasma membrane [5,6], LPPs also associate with membrane microdomain rafts and caveolae [5-7]. Ovarian carcinoma cells secrete a high concentration of S1P and LPA [8,9]. Accordingly, overexpressing sphingosine kinase 1 (Sphk1), one of the main enzymes that catalyzes the formation of S1P, results in increased tumorigenic activity in the *Min *mouse model of intestinal tumor progression [10]. Additionally, elevated expression of LPPs has been postulated to reduce the level of S1P and its structural homologs, thereby down-regulating cell signaling. In support of this notion, *in vitro *experiments have provided evidence for the ability of LPPs to down-regulate the activity of extracellular signal-regulated kinase (Erk)1/2 and induce death of ovarian carcinoma cells [11,12]. However, LPP3 has been shown to be highly expressed in sprouting endothelial cells and can act as a cell associated integrin ligand [13-15]. Accordingly, we have shown that an antibody against the extracellular domain of LPP3 inhibits cell-cell interactions and angiogenesis *in vitro *[14-16]. In a separate study, we have also shown that LPP3 is required for tumor adaptation and β-catenin signaling [17,18]. These observations provided us with the initial impetus to carry out this study on the role of LPPs in the acquisition of neoplastic cellular behavior.

In addition to binding to E-cadherin, a key role for β-catenin has been established in Wnt (*wingless*) signaling. The activation of canonical Wnt signaling promotes the translocation of β- catenin to the nucleus, where it acts as a co-activator for the transcription factor T-cell factor/lymphocyte enhancer binding factor (TCF/LEF-1) complex. Increased Wnt/β-catenin signaling has been linked to cancer cell proliferation, stem cell self-renewal, and angiogenesis. In this regard, β-catenin signaling is often shown to up-regulate transcription of the CYCLIN-D1 protein. CYCLIN-D1 is a key regulator of cell cycle progression, the increased expression of which is a fundamental characteristic of tumor cell proliferation. Although *Lpp3 *is essential for vasculogenesis, aspects of embryonic development and Wnt signaling [19], the function of LPP3 during pathological processes, including tumor growth, remains unknown. A few reports have focused on the role of LPPs in angiogenesis [14,16-19], but none has reported the expression patterns of LPP3 in human primary tumors or tumor cell lines. Importantly, the association of increased LPP3 expression with cell transformation and tumorigenesis has not been reported. To test whether LPP3 regulates tumor cell behavior, we knocked down *LPP3 *and investigated glioblastoma cell proliferation and tumor formation; conversely, we forced expression of *LPP3 *in LPP3-deficient human colon carcinoma (SW480) cells to address the hypothesis that LPP3 potentiates cell proliferation and tumor growth.

## Results

### Expression of LPP3 Protein in a Subset of Primary Tumors

**Figure 1:**
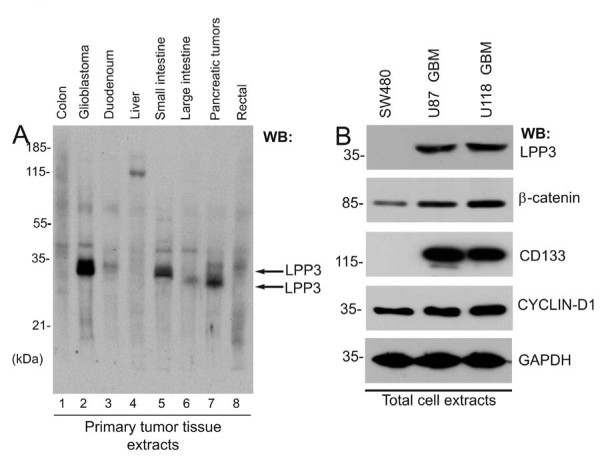
**Expression of LPP3 in primary tumors**. **(A) **Western blot was probed with an anti-LPP3-C-cyto antibody. Each lane contained 10 μg total protein prepared from primary human tumor tissues. LPP3 protein is indicated by two black arrows. **(B) **Tumor line cell extracts were analyzed by WB with the indicated antibodies. All blots shown are representative of those obtained from at least three separate experiments with similar results.

**Figure 2:**
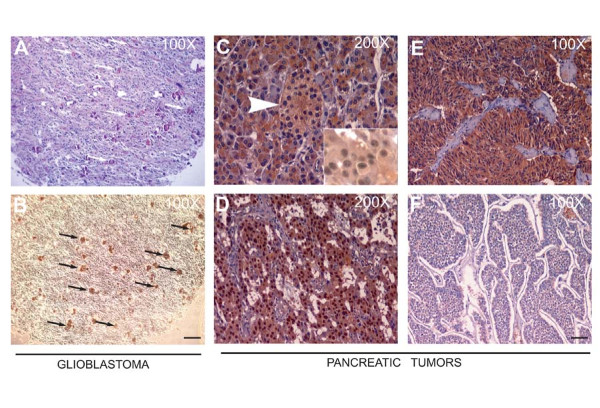
**Immunohistochemistry of primary tumors**. **(A) **A representative serial section of human glioblastoma was stained with hematoxylin and eosin (H&E), white arrows indicate microvascular proliferation, and **(B) **black arrows indicate anti-LPP3-RGD positive glioblastoma cells. Scale bars, 100 μm. **(C) **Anti-LPP3 staining in normal pancreas show cytosolic expression in both endocrine (Islet of Langerhans-arrow) and exocrine components. **(D, E) **In pancreatic endocrine tumors, 65% of the tumors show cytosolic staining. Inset: Nuclear localization of LPP3 is also detected in 40% of cases. **(F) **An example of LPP3-negative tumor cells. All experiments are representative of those obtained in at least three separate experiments, with similar results.

Expanding upon our previous reports [13,14], we assessed the expression of LPP3 protein in primary tumors of various histotypes. The specificity of anti-LPP3-C-cyto has been addressed in our previous publications [13-18]. Using this antibody, we noted increased expression of LPP3 in glioblastoma (GBM), small intestine, and pancreatic tumor samples (Figure 1A). LPP3 expression was low in colon, duodenum, large intestine, and rectal tumor samples. LPP3 expression in liver tumors was not detectable, except for a non-specific high molecular weight species (~125 kDa; Figure 1A). In keeping with the survey, cell lines of different histotypes corresponding to high and low/undetectable LPP3 levels were chosen for further analysis. In primary tumors, anti-LPP3-C-cyto antibody primarily detected one immature 32-33 kDa (indicated by two black arrows) and one mature 38-40 kDa polypeptide species (Figure 1A). Maturation of LPP3 protein requires N-glycosylation [14,16]. Since primary tumors are not likely synchronized, these cells could express both mature and immature forms of LPP3 polypeptides. The human glioblastoma cell lines, U87 and U118, expressed LPP3, whereas SW480 human colon tumor cells did not (Figure 1B, top panel). Compared to SW480 cells, U87 and U118 cells expressed appreciably higher levels of β-catenin and CYCLIN-D1 (Figure 1B). Interestingly, SW480 did not express a detectable level of CD133 (a marker of stem cells), while U87 and U118 cells expressed CD133 (Figure 1B). We also analyzed an array of 63 glioblastoma tissue sections that were glial fibrillary acidic protein (GFAP) positive; of 63 sections analyzed, 11 sections demonstrated anti-LPP3-RGD antibody immunoreactivity (Figures 2A and 2B). We expanded the analysis to a series of well-characterized primary pancreatic tumors, in which we detected cytosolic expression of LPP3 in both endocrine and exocrine normal pancreas, and in 41/63 pancreatic tumors of neuroendocrine origin with evidence of altered subcellular (nuclear) localization in 40% of the cases (Figures 2C-F). These data suggest that LPP3 expression is increased in a subset of solid tumors and in U87 and U118 glioblastoma tumor cell lines.

### LPP3-Knockdown Reduces Glioblastoma Tumor Cell Proliferation and Migration

**Figure 3:**
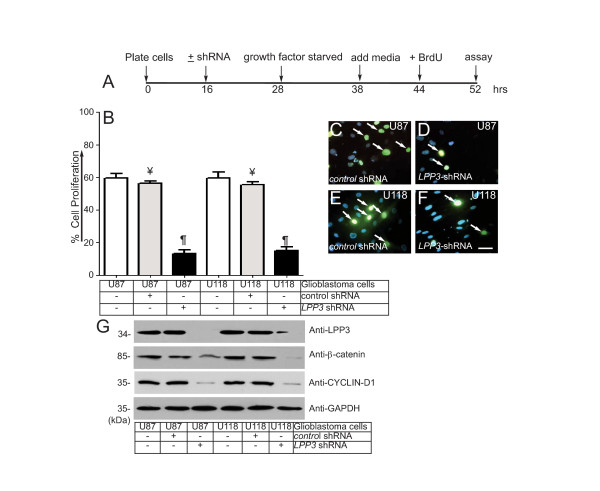
**LPP3 regulates glioblastoma cell proliferation**. **(A) **The time line indicates when glioblastoma cell lines were plated, shRNA (retrovirus) mediated knockdown was conducted, cells were growth factor and serum starved, fresh medium was added, BrdU (1.0 μg/ml) was added, and the proliferation assay was performed. **(B) **Quantification of glioblastoma cell proliferation assay. The data represent the mean ± s.e.m. n = 5-7 from five to seven independent experiments, ¥ P < 0.05 *vs. *control untreated; ¶ P < 0.05 *vs. *control shRNA treated group. **(C-F) **Representative images of the BrdU assay. Arrows indicate BrdU positive cells. Scale bar, 150 μm. **(G) **The efficiency of knockdown of *LPP3 *was assessed by western blotting with the indicated antibodies. Figure 3G (top panel, last lane, anti-LPP3), a partial band is likely produced by over flow from the neighboring well. All experiments are representative of those obtained in at least three separate experiments with similar results.

The acquisition of increased proliferative and migratory phenotypes is a hallmark of neoplastic cells. Given that β-catenin and CYCLIN-D1 are known regulators of tumor cell proliferation and migration, we examined the relationship between LPP3 expression and β-catenin and CYCLIN-D1 in U87 and U118 glioblastoma cell proliferation and migration. The timeline of this experiment is shown in Figure 3A. We observed that LPP3 depletion significantly inhibited the proliferation of both U87 and U118 cells, whereas control shRNA had no effect (Figure 3B). Representative images of the BrdU assay are shown (Figure 3C-F). *LPP3 *knockdown decreased β-catenin and CYCLIN-D1 expression in both U87 and U118 cells, whereas it had no effect on GAPDH levels (Figure 3G).

**Figure 4:**
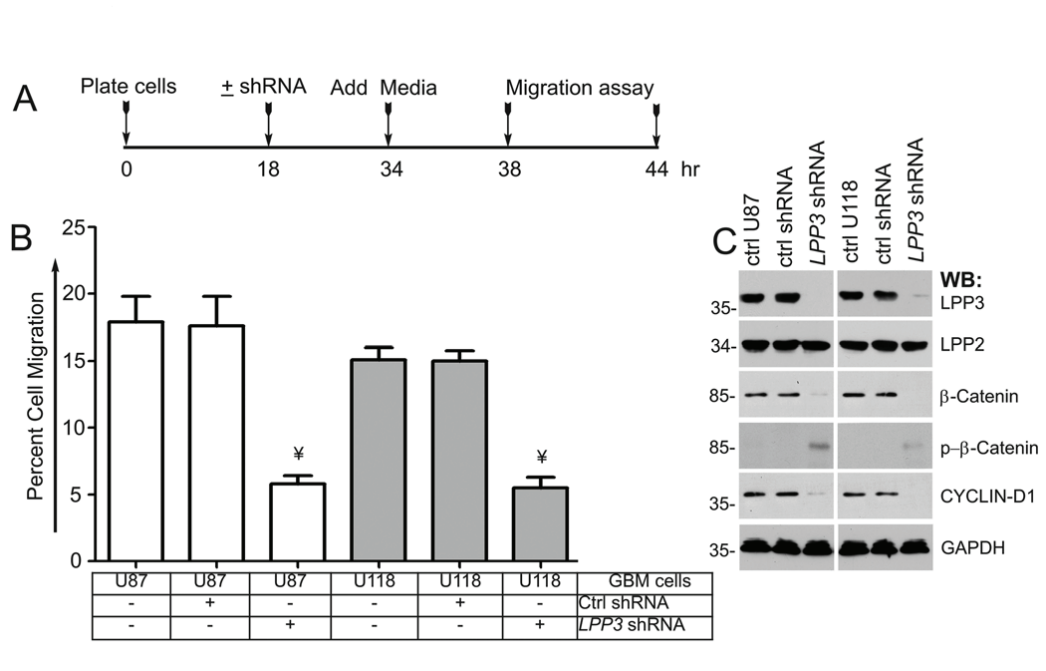
**LPP3 regulates glioblastoma cell migration**. **(A) **The time line indicates when glioblastoma cell lines were plated, shRNA (retrovirus) mediated knockdown was conducted, medium was added, and the migration assay was performed as described in materials and methods. **(B) **Quantification of glioblastoma cell migration through the Boyden chamber. The data represent the mean ± s.e.m. n = 5-7, ¥ P < 0.05 vs. control shRNA treated group. **(C) **Efficiency of knockdown of LPP3 was assessed by western blotting with the indicated antibodies. All experiments are representative of those obtained in at least three separate experiments with similar results.

To examine the effect of *LPP3 *knockdown on cell migration, we carried out a cell migration assay using a chemotactic Boyden chamber (Figure 4). The timeline of this experiment is shown in Figure 4A. *LPP3 *knockdown reduced the migration of both U87 and U118 cells in the Boyden chamber assays, whereas control shRNA had no effect (Figure 4B). The efficiency of *LPP3 *knockdown was close to 100% in U87 cells and was 80-90% in the U118 tumor line (Figure 4C). *LPP3 *knockdown had no effect on LPP2 protein level (Figure 4C), but it increased the level of phosphorylated β-catenin species. Thus, the β-catenin level decreased (Figure 4C), and the level of CYCLIN-D1 was concomitantly decreased in LPP3-depleted cells. These data show that *LPP3 *knockdown reduced cell proliferation and migration by down-regulating β-catenin and CYCLIN-D1 activities.

### LPP3 Knockdown Reduced U87 and U118 Glioblastoma Tumor Growth

**Figure 5:**
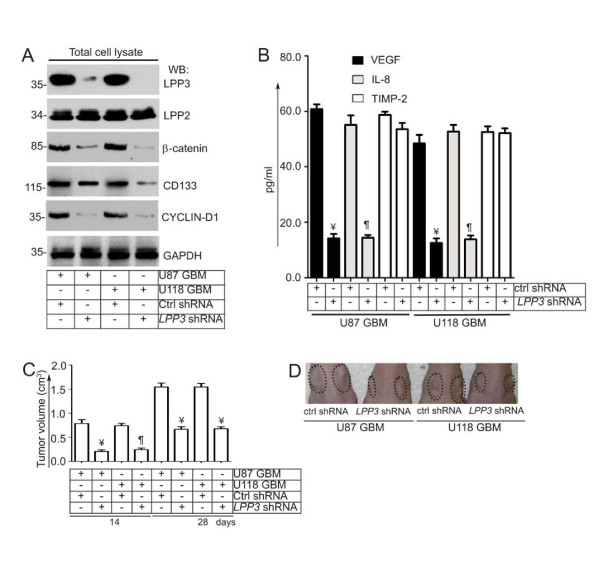
**LPP3 regulates glioblastoma tumor growth**. **(A) **Stable clones of U87 and U118 were selected and efficiency of knockdown was determined by western blotting with the indicated antibodies. *LPP3*-knockdown decreased total β-catenin, CYCLIN-D1, and CD133 proteins, whereas it had no effect on LPP2 or GAPDH. **(B) **Conditioned media were collected and subjected to ELISA for VEGF, IL-8, and TIMP-2. *LPP3*-knockdown in U87 and U118 cells reduced the concentration of VEGF and IL-8, whereas TIMP-2 was unaffected. **(C) **In the xenograft assay, mice receiving cells that expressed control shRNA showed increased tumor growth (n = 12, ¥ P < 0.05) after 14 and 28 days. *LPP3*-knockdown reduced U87 and U118 tumor growth (n = 12, ¶ P < 0.05). **(D) **Representative images from the xenograft assay. Dotted circles indicate the location of tumor implants.

Next, we performed *LPP3 *knockdown in U87 and U118 cell lines and tested their ability to grow in a xenograft experiment (Figure 5). First, we assessed the effect of LPP3 depletion on β-catenin and CYCLIN-D1 levels by western blot analysis (Figure 5A). LPP3 depletion decreased total β-catenin, CYCLIN-D1, and CD133 protein, whereas it had no effect on LPP2 or GAPDH protein levels (Figure 5A). To examine the effect of LPP3 depletion in U87 and U118 cells, conditioned media were collected and subjected to ELISA for the presence of vascular endothelial growth factor (VEGF) and interleukin-8 (IL-8), both of which are known downstream targets of canonical Wnt signaling. We included tissue inhibitor of metalloproteinases-2 **(**TIMP-2) as a control (Figure 5B). LPP3-depleted U87 and U118 cells produced significantly decreased concentrations of VEGF and IL-8, whereas TIMP-2 levels remained unchanged (Figure 5B). In the xenograft experiment, mice receiving U87 and U118 that had been treated with control shRNA showed significant tumor growth after 14 and 28 days (Figure 5C). In contrast, LPP3-depleted U87 and U118 cells showed reduced tumor growth (Figure 5C). Representative images of the xenograft experiment are shown (Figure 5D) and suggest that LPP3-depletion in glioblastoma tumors reduces tumor growth by dampening β-catenin and CYCLIN-D1 activity.

### Elevated Expression of LPP3 Potentiates SW480 Growth in Xenograft Assay

**Figure 6:**
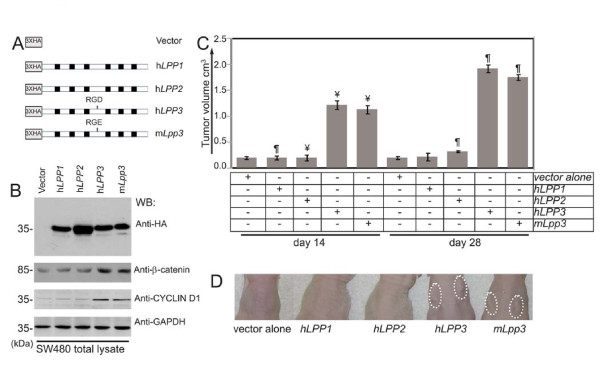
**Forced expression of LPP3 potentiates SW480 tumor growth in the xenograft assay**. **(A) **pLNCX2-based retroviral constructs expressing vector alone, hLPP1, hLPP2, hLPP3 or mLpp3 cDNAs. **(B) **The comparable levels of hLPP1, hLPP2, hLPP3 and mLpp3 proteins were confirmed by western blotting with anti-HA antibody. **(C) **Xenograft assay: there was minimal tumor growth after 14 and 28 days in mice receiving LPP3-deficient SW480 cells (2 × 10^4 ^tumor cells/site) that expressed vector alone, hLPP1, or hLPP2 constructs. In contrast, tumor volume was significantly increased in mice receiving SW480 cells that expressed hLPP3 or mLpp3 (n = 12, ¥ P < 0.01 vs. hLPP1 group; n = 12, ¶ P < 0.005 vs. hLPP1 group) at 14 and 28 days.

To test the ability of LPP3 to regulate tumor growth, we used LPP3-deficient SW480 colon tumor cells to investigate the relationship between elevated expression of LPP3 and tumor growth. SW480 harbors two mutant alleles of the *APC *gene [20], which results in the stabilization of β-catenin. First, we prepared retroviral (pLNCX2) constructs that expressed the indicated *LPPs *(Figure 6A) and generated stable clones. Because results obtained from over-expression experiments can be ambiguous, only clones that displayed the lowest expression levels were selected. Clones expressing comparable levels of hLPP1, hLPP2, hLPP3 and mLpp3 proteins were selected based on the results of western blot analysis (Figure 6B). β-catenin was not increased in SW480 cells that expressed *hLPP1 *or *hLPP2*. In contrast, the stability of β-catenin and CYCLIN-D1 protein was increased in SW480 cells that expressed h*LPP3 *or m*Lpp3 *(Figure 6B). As athymic nude mice do not mount an immunogenic response to xenografts, we investigated the role of elevated LPP3 expression of in SW480 cells using these mice. We observed minimal tumor growth after 14 and 28 days in mice that received LPP3-deficient SW480 cells expressing vector alone or *hLPP1 *or *hLPP2 *(2 × 10^4 ^tumor cells/site) (Figure 6C). In contrast, SW480 cells expressing *hLPP3 *or *mLpp3 *grew significantly (n = 12, ¶ P < 0.01 *vs. hLPP1 *group) within 28 days. Closer examination of *hLPP3- *and *mLpp3*-expressing tumors showed a vascularized tumor with localized hemorrhaging, indicative of aggressive tumor growth (Figure 6D). In contrast, *hLPP1*, *hLPP2*, *hLPP3 *or *mLpp3 *did not transform NIH-3T3 cells in culture (data not shown). Together, these data strongly suggest that LPP3 is not a tumor promoting factor, but it amplifies β-catenin signaling and CYCLIN-D1 activity to potentiate SW480 growth.

### Both the RGD and the Lipid Phosphatase Domains of LPP3 are required for SW480 Tumor Growth

**Figure 7:**
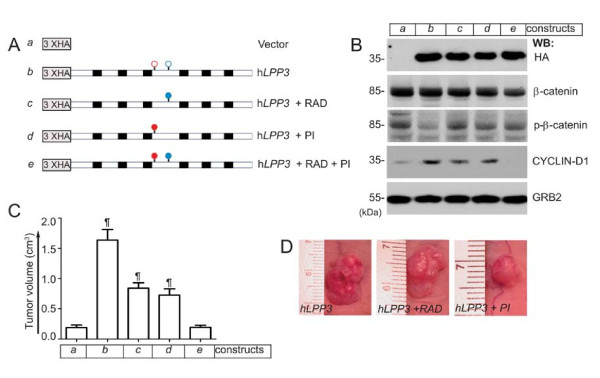
**Domains involved in the regulation of SW480 tumor growth**. **(A) **Schematics of pLNCX2-based retroviral constructs (a-e). Empty red and blue circles indicate the relative positions of lipid phosphatase and RGD cell adhesion domains, respectively. Filled red and blue circles indicate phosphatase-inactive (PI) and RAD (adhesion defective) domains, respectively. Black boxes represent transmembrane segments. Three copies of hemagglutinin (HA) epitopes were fused to the N-terminal and in-frame to the open reading frame of the cDNAs. **(B) **Equivalent protein expression and phosphorylation states of β-catenin and CYCLIN-D1 proteins were analyzed by immunoblotting with anti-HA, anti-β-catenin, anti-p-β-catenin, and anti-CYCLIN-D1 antibodies. Equal loading of proteins across the lanes were determined with the anti-GRB-2 antibody. All blots are representative of those obtained in at least three separate experiments with similar results. **(C) **LPP3 expression potentiates SW480 tumor growth. Nude mice were injected subcutaneously with SW480 cells (~ 2 × 10^4^) expressing the indicated constructs. After 21 days, the tumor outgrowths were measured and photographed. (n = 12, ¶ P < 0.005 vs. vector alone group). **(D) **Representative pictures of primary tumors in nude mice injected with SW480 cells expressing the indicated constructs.

To elucidate which domain of LPP3 regulates β-catenin stability and CYCLIN-D1 expression, thereby potentiating SW480 tumor growth, we prepared pLNCX2-based vector alone (construct-a), wild-type (construct-b, h*LPP3*), adhesion defective mutant (construct-c, h*LPP3*-RAD), phosphatase-inactive mutant (construct-d, h*LPP3*-PI), and double mutant (construct-e, h*LPP3*-RAD + PI) constructs (Figure 7A). Stable colonies of SW480 cells were prepared and then selected for equivalent protein expression by western blotting with anti-HA antibodies (Figure 7B, top panel). There was no anti-HA immunoreactive species in the pLNCX2-based vector alone (construct-a). We also determined total β-catenin levels, the phosphorylation status of β-catenin, and CYCLIN-D1 levels prior to performing the tumor implantation experiment (Figure 7B). We observed decreased β-catenin phosphorylation in SW480 cells that expressed hLPP3 (construct-b), whereas there was no major change in the total level of β-catenin in these cells (Figure 7B). For the xenograft assay, only those clones that displayed low expression levels were used. SW480 cells expressing the indicated constructs were then injected into nude mice subcutaneously (s.c.), and tumor volume was examined after 21 days (Figure 7C). Tumor growth in control mice (construct-a) was minimal (Figure 7C). However, we observed increased tumor growth in mice that received SW480 cells expressing h*LPP3*, but this effect was significantly reduced in mice that received cells expressing h*LPP3*-RAD and h*LPP3*-PI. Importantly, the tumor volume in mice receiving h*LPP3*-RAD + PI was similar to that in mice receiving vector alone. Representative images of this experiment are shown in Figure 7D.

## Discussion

In this report we showed that LPP3 was constitutively expressed in a subset of primary tumors and in U87 and U118 cells, whereas it was undetectable in a human adenocarcinoma E-cadherin deficient SW480 cell line. Because Wnt/β-catenin signaling is often constitutively active in many neoplastic cells, we posited that constitutive expression of LPP3 in glioblastoma cells may serve as a link in the acquisition of proliferative, invasive, and metastatic phenotypes. Glioblastoma is an aggressive type of brain tumor [21]. The lethal behavior of glioblastoma is characterized by its ability to invade quickly, usually throughout the normal brain parenchyma, and to evade apoptosis, thereby rendering it incurable with traditional chemotherapy and radiation [21,22]. In this regard, neural stem cells have been shown to differentiate into endothelial cells. In fact, glioblastoma cells might have the ability to transition into endothelial-like cells [23-25]. Although LPP3 function is restricted to endothelial cells, LPP3 could have neural function as well. Thus, herein we addressed the relationship between increased LPP3 expression and glioblastoma tumor growth *in vitro *and *in vivo*.

Increased proliferation and migration are key hallmarks of tumor cells. Beyond binding to VE- or E-cadherin, β-catenin transduces Wnt signaling to serve as a transcriptional co-activator that alters the expression of target genes involved in cell proliferation, survival, and differentiation. CYCLIN-D1 is one of the key targets of the Wnt/β-catenin/LEF-1 transcriptional machinery that induces the progression of cells through the G1 phase of the cell cycle. β-catenin and CYCLIN-D1 are major regulators of tumor cell proliferation and migration. LPP3 depletion reduced β-catenin and CYCLIN-D1 proteins, thereby impairing U87 and U118 glioblastoma cell proliferation and migration. Reduction of β-catenin species may have also been due to the increased phosphorylation of β-catenin in these cells. These results strongly suggest that *LPP3 *knockdown impedes cell proliferation and migration by reducing β-catenin and CYCLIN-D1 levels.

Xenograft experiments demonstrate that *LPP3 *knockdown decreased the levels of not only β-catenin and CYCLIN-D1, but also CD133 (a marker of stem-like cells), VEGF and IL-8. Expression of CD133 by U87 and U118 cells is consistent with recent reports [23-27]; notably, it has been proposed that CD133+ glioblastoma tumor cells give rise to tumor endothelium cells [23,24]. Therefore, our results suggest a key role of LPP3 in the generation of CD133+ cells. VEGF and IL-8 are a pro-angiogenic growth factor and cytokine, respectively. Because VEGF and IL-8 are activated by β-catenin signaling [26], it was not surprising that U87 and U118 glioblastoma cells expressed high levels of these two angiogenic factors [26]. Importantly, *LPP3 *knockdown significantly decreased levels of VEGF and IL-8, but not of TIMP-2, in these cells. Whether *LPP3 *knockdown affects differentiation of glioblastoma tumor cells into tumor endothelium will require detailed investigation. However, our data suggest that *LPP3 *knockdown also affects the level of CD133 protein and secretion of factors that are required for tumor neovascularization.

In the converse experiment, we determined the impact of elevated LPP3 expression in LPP3-deficient SW480 colon tumor cells and performed a xenograft assay using athymic nude mice. In this regard, tumorigenic SW480 cells harbor two mutant alleles of *Adenomatous Polyposis Coli *(*APC*^mut/mut^) gene, but normal *β-catenin *[20]. Accordingly, we have demonstrated that the forced expression of h*LPP3 *and m*Lpp3 *in SW480 cells potentiated tumor growth and metastasis in the xenograft assay, whereas h*LPP1 *and h*LPP2 *had no effect. Importantly, the forced expression of *LPP3 *decreased the basal phosphorylation status of β-catenin, thereby enhancing the stability of β-catenin species. Altogether, these data suggest that the increased proliferation and tumor growth are LPP3-specific and mediated by increased β-catenin and CYCLIN-D1 functional activity.

To define the minimal functional domains of LPP3 involved in the potentiation of tumor growth, we generated retroviral constructs carrying functional mutations. Stable clones of SW480 cells expressing these constructs were implanted in nude mice. h*LPP3 *induced robust tumor growth, whereas h*LPP3*-RAD or the h*LPP3*-PI supported SW480 tumor growth at a diminished rate. However, the tumor volume was significantly reduced (as in control cells) in mice receiving SW480 cells expressing the h*LPP3*-RAD + PI construct. These data suggest that the LPP3 protein has at least two functional domains, the lipid phosphatase and the LPP3-RGD cell adhesion domains. The forced expression of *LPP3 *in NIH-3T3 fibroblast cells did not transform these cells (data not shown), suggesting that the LPP3 is not tumorigenic, but in combination with genetic instability such as the mutation of *APC*, elevated expression of LPP3 potentiates tumor progression by stimulating β-catenin and CYCLIN-D1 transcriptional activity.

## Conclusions

One long-held view is that LPP3 down-regulates cell signaling by virtue of its ability to dephosphorylate S1P and its structural homologs. In contrast, our data show the ability of LPP3 to potentiate tumor growth by amplifying β-catenin and CYCLIN-D1 activities. Therefore, these unexpected set of results implicate LPP3 as a potential target for inhibiting the growth of glioblastoma and perhaps other tumors that express high levels of LPP3.

## Methods

### Cells and Reagents

Human glioblastoma U87 and U118 cell lines were purchased from the American Type Culture Collection (Manassas, VA). E-cadherin-deficient (ECD) human colon adenocarcinoma SW480 (*APC*^mut/mut^) cells were a kind gift of Dr. Cara Gottardi (Northwestern University, Chicago, IL). Preparation of rabbit anti-LPP3 polyclonal antibodies (pAbs) has been described previously [14,15]. Mouse anti-VCIP/LPP3 (39-1000) monoclonal (mAb) and anti-LPP2 pAb were purchased from Invitrogen (Carlsbad, CA) and Exalpha Biologicals, Inc (Shirley, MA), respectively. Anti-CYCLIN-D1 (2978), rabbit anti-phospho-β-catenin (Ser33, Ser37, and Thr41) and rabbit anti-CD133 polyclonal antibodies were purchased from Cell Signaling Technology, Inc. (Denver, MA). Anti-β-catenin (SC-7963), anti-GAPDH (SC-51906) and anti-GRB2 pAbs were purchased from Santa Cruz Biotechnology (Santa Cruz, CA). Secondary antibodies were purchased from Promega (Madison, WI).

### Immunohistochemistry

Glioblastoma tissue arrays were purchased from U.S. Biomax Inc. (Rockville, MD). Paraffin-embedded tissues were collected from normal pancreas and 63 pancreatic endocrine tumor (PET) affected patients resected at the University Hospital of Verona, Italy. Informed consent for the analysis of normal and neoplastic tissues was obtained from patients in accordance with the approval of the Verona University and Hospital Trust's Ethical Committee. Four-micrometer-thick sections were prepared. Antigen retrieval was performed in 10 mM citrate buffer (pH 6.0) and heated in a microwave at 360 W for 20 min. Endogenous peroxidase and non-specific sites were blocked by incubation, respectively, with 3% H_2_O_2 _and 10% goat serum at room temperature. Sections were then incubated with a rabbit IgG or a rabbit anti-LPP3-RGD antibody (5 μg/ml) for 1 h and 30 min at room temperature, washed three times with PBST (PBS/0.2% Tween-20), and incubated for 30 min with anti-rabbit conjugated peroxidase (DakoEnVision+^®^, Peroxidase, Mouse and Rabbit Ready-to-use). After 3 washes with PBST, diaminobenzidine (DAB) (Vector Biolaboratories, SK-4100) was used as a peroxidase substrate for 10 minutes at room temperature.

### Recombinant cDNA constructs, cell culture, retroviral transduction, and stable cell line generation

pLNCX2 and the amphotropic packaging cell line, HEK293 (human kidney fibroblasts), were purchased from BD Biosciences (San Jose, CA). Generation of recombinant cDNA constructs in pLNCX2 retroviruses have been described previously [14,15,18]. Control and *LPP *shRNA retroviral constructs were purchased from Origene (Rockville, MD). All transfection experiments have been previously described [14,15,18]. Stable clones were selected by western blotting, and LPP expression levels were determined by immunoblotting as described previously [14,15,18].

### Cell extraction and immunoprecipitation

Cell extracts were prepared using 20 mM Tris (pH 7.5), 150 mM NaCl, 1% Triton X-100, 0.25% NP-40, and 1 mM EDTA buffer. All biochemical experiments, including western blot (WB) analyses, were performed as described previously [14,15,18,28]

### Cell migration assay

Migration assay was carried out as described previously [18]. In brief, modified transwell Boyden chambers (8 μM) were used for chemotactic cell migration. Human U87 and U118 cell were plated overnight in complete media, next day cells at 60% density were infected with control- or *LPP3*-shRNA retroviral particles for 12 hours. Media containing viral particles were removed and replenished with media with containing insulin, transferrin and selenium (hereafter called Migration Assay Media, MAM) without serum. After 4 hours, cells detached with 2 mM EDTA, pH 7.4, pelleted, washed twice with PBS (pH 7.5), and resuspended in MAM. The upper chamber was filled with 400 μl of MAM containing ~1 × 10^4 ^cells, and the lower chamber was filled with 500 μl of MAM, no chemo-attractant was used. Cells were incubated for 6 h at 37°C in a CO2 incubator. After 6 hours, inserts were retrieved and cells in the upper chamber removed with q-tips, while cells migrated to the lower face of the membrane were fixed and stained with 0.5% crystal violet. Excess dye was washed with running tap water, membranes were then examined using a phase-contrast microscope. At least seven 200 × magnification fields were selected for scoring. Cell migration assay was repeated 3 times with each trial being performed in triplicate.

### Enzyme-linked Immunosorbent Assay (ELISA)

ELISAs were performed as described previously [18,28]. To monitor the secretion of VEGF, IL-8, and TIMP-2, U87 and U118 cell lines were left in medium containing 5 μg/ml puromycin for 24 hours. Then, the cell culture media was collected, centrifuged and subjected to ELISA according to the manufacturer's recommendations (Research Diagnostics and R & D Systems). The ELISA detection limit for each factor ranged from 6 pg/ml to 1600 pg/ml, and the intra- and inter-assay variations were 4.1-6.2% and 6.5-10%, respectively.

### Xenograft assay

All experiments described in this study were carried out according to the University of Illinois (UIC) Animal Care Committee (ACC) and NIH guidelines. Eight-week old female (~25 g) athymic nude mice from Jackson Laboratory (Bar Harbor, ME) were used for this study. SW480 cells expressing vector (pLNCX2) alone and various *LPP *constructs were trypsinized, resuspended and washed with sterile PBS, pH 7.4. After resuspension in PBS, pH 7.4, cells were passed through a 100 μM cell strainer and enumerated using a hemocytometer. Cells (2 × 10^4 ^in 150 μl) were subcutaneously (s.c.) injected into each of 12 animals (n = 12) per group. Tumor growth was monitored for 21 or 28 days. One mouse from each group was selected randomly, anesthetized, photographed, and sacrificed and the tissues were recovered for further analysis.

### Statistics

Analysis of variance (ANOVA) was used for statistical analyses and values of *P *< 0.05 were considered statistically significant. Data represent means ± s.e.m. All analyses were performed using GraphPad Prizm 5.0 software (La Jolla, CA).

## Competing interests

The authors declare that they have no competing interests.

## Authors' contributions

IC and JOH performed transfection experiments, shRNA mediated knockdown, biochemical and xenograft assays. EEK and CS performed the immunohistochemical studies on human tumor samples, analyzed and interpreted data as well as carried out statistical analyses. KKW designed the study, analyzed and interpreted data, prepared final figures, and drafted the manuscript. All authors read and approved the manuscript.
